# Inhibition of Sodium Glucose Cotransporters Improves Cardiac Performance

**DOI:** 10.3390/ijms20133289

**Published:** 2019-07-04

**Authors:** Álvaro García-Ropero, Ariana P. Vargas-Delgado, Carlos G. Santos-Gallego, Juan J. Badimon

**Affiliations:** 1 Atherothrombosis Research Unit, Mount Sinai Heart, Icahn School of Medicine at Mount Sinai, New York, NY 10029, USA; 2 Cardiology Department, Imperial College London, The Royal Brompton and Harefield Hospital, London 6W3 6NP, UK; 3 Instituto Ecuatoriano del Corazón IECOR, Guayaquil 090513, Ecuador

**Keywords:** sodium-glucose cotransporter, ischemia reperfusion injury, heart failure, cardiac metabolism

## Abstract

The sodium-glucose cotransporter (SGLT) inhibitors represent a new alternative for treating patients with diabetes mellitus. They act primarily by inhibiting glucose reabsorption in the renal tubule and therefore, decreasing blood glucose levels. While little is yet known about SGLT subtype 1, SGLT2 inhibitors have demonstrated to significantly reduce cardiovascular mortality and heart failure hospitalizations. This cardioprotective benefit seems to be independent of their glucose-lowering properties; however, the underlying mechanism(s) remains still unclear and numerous hypotheses have been postulated to date. Moreover, preclinical research has suggested an important role of SGLT1 receptors on myocardial ischemia. Following acute phase of cardiac injury there is an increased activity of SGLT1 cotransport that ensures adequate energy supply to the cardiac cells. Nonetheless, a long-term upregulation of this receptor may not be that beneficial and whether its inhibition is positive or not should be further addressed. This review aims to present the most cutting-edge insights into SGLT receptors.

## 1. Introduction

Optimal myocardial performance relies upon incessant generation and supply of ATP molecules to the contractile apparatus. To generate this energy, the heart utilizes a variety of different metabolites including glucose, fatty acids (FA), ketone bodies (KB), and lactate among others. Interestingly, cardiac cells are capable of shifting energy dependence among these substrates depending on their availability and myocardial workload changes (also termed “metabolic flexibility”) [1]. Numerous disturbances affecting both metabolic pathways and substrate flexibility have been associated with contractile impairment [2,3] and novel targeted-therapies are now under investigation. This indeed, represents a promising alternative for such patients, considering that the leading cause of death worldwide is heart disease [4]. 

The novel antidiabetic drug family of sodium-glucose cotransporter (SGLT) inhibitors has been found to significantly reduce cardiac mortality in patients with type 2 diabetes mellitus (T2DM) [5,6,7]. Furthermore, SGLT2 inhibitors (SGLT2i) protect renal function in diabetic patients in a non-related glucose-lowering fashion [5,6,8,9,10], but the definitive mechanism(s) remains unclear. Among all SGLT subtypes, SGLT1 and SGLT2 are the most abundant receptors, but only the former is expressed in cardiac cells [11]. Whereas little is yet known about SGLT1 inhibition, most of the literature has focused on studying SGLT2 properties and the impact of its blockage in T2DM patients [12]. Interestingly, emergent data suggest a potential role of SGLT1 receptor on ischemic heart disease (IHD). Therefore, in this review we aim to present the most cutting-the-edge literature regarding the impact of both SGLT1 and SGLT2 inhibition on myocardial damage and cardiac-cell performance.

## 2. Cardiac Metabolism and Substrate Bioenergetics

### 2.1. Normal Cardiac Metabolism

Human hearts obtain energy from two sources, adenosine triphosphate (ATP) (~5 μmol/g wet weight) the primary molecule to act as fuel in all cellular processes, and phosphocreatine (PCr) (~8 μmol/g wet weight) which acts more as an energy reserve [1]. Cardiomyocytes contain numerous mitochondria, an organelle that generates over 95% of ATP through oxidative phosphorylation. The remaining 5% is produced by both glycolysis in the cytoplasm, and a minimum part by the Krebs cycle [13,14]. PCr can be obtained from ATP’s high-energy phosphate bond, which is transmitted to creatine via interaction with creatine kinase (CK) enzyme. Due to its low molecular weight, PCr is capable to cross the membrane into the cytosol easily, and to generate more ATP molecules from precipitated adenosine diphosphate (ADP) by CK [15].

**Figure 1:**
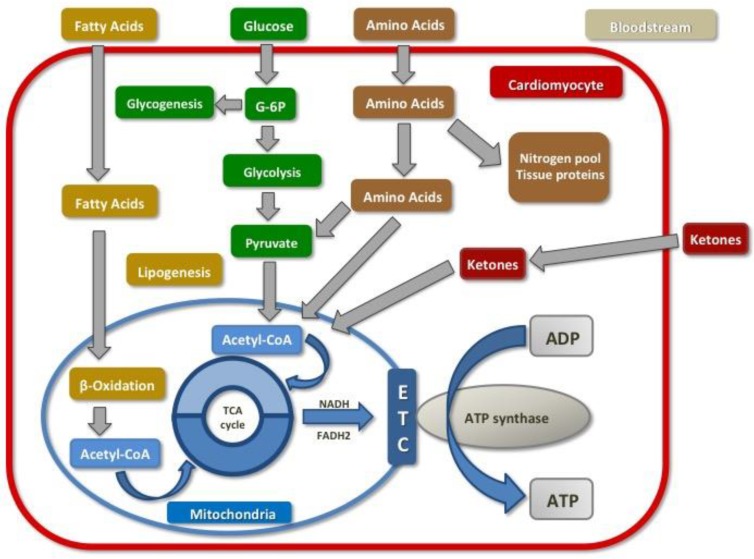
Figure 1 represents normal cardiac metabolism and bioenergetics.

The myocardium requires high amounts of energy to meet the constant demand of sarcomere proteins. In order to maintain this, the cardiac cells rely on substrate flexibility which allows them to switch from one source of energy to another one depending on cardiac workload and metabolite availability [14]. During a fasting state, the main source for cardiac ATP (up to 70% of total production) comes from FA oxidative phosphorylation, 20% from glucose and the remaining from amino acids (AA), lactate and KB consumption [16]. In the fed state, with the presence of high blood carbohydrates levels, the principal substrate is glucose. Furthermore, during long-standing exercise, lactate becomes the main source of ATP supply to the myocardium since cardiac cells are capable to switch among different substrates [14] (Figure 1).

### 2.2. The Failing Heart and Diabetic Cardiomyopathy: Metabolic Alterations

When the heart fails to supply enough energy (ATP molecules) to meet energy demands (ATP consumptions, an impairment in cardiac performance may be induced [14]. This phenomenon is worsened given the fact that the human heart only storages ATP for around 5–10 s. In order to survive this ordeal, the myocardium switches cardiac metabolism back to a fetal model wherein FA oxidation decreases and carbohydrate metabolism (both aerobic and anaerobic) becomes the main source of energy.

In normal hearts, Acetyl-CoA, a common denominator from different metabolic pathways, enters in the Krebs cycle within the mitochondria to experience oxidative phosphorylation and to obtain ATP. In the failing heart, there is an increased glycolysis and upregulation in anaplerosis pathways to provide Acetyl-CoA molecules for the Krebs cycle. This metabolic switch is based on the “Randle cycle” regulation that postulate an increased glucose oxidation when glucose availability exceeds FA and vice versa [17]. This change in myocardial utilization from FA towards glucose is oxygen-efficient since glucose oxidation consume less oxygen molecules as compared to FA oxidation, and thus is positive in the short term; in fact, the P/O ratio (the number of ATP molecules produce per oxygen molecule) is higher for glucose (P/O = 2.58) than for FA (P/O = 2.33). However, this metabolic switch fails to supply enough energy given the fact that FA consumption produces 105 molecules of ATP in comparison with only 31 molecules of ATP from glucose consumption, hence this metabolic switch is deleterious in the long term and thus gives birth to the concept “heart failure begets heart failure (HF)”.

A different mechanism has been postulated for hyperglycemic states and diabetic cardiomyopathy. As there is reduced glucose uptake and utilization due to insulin resistance, FA oxidation increases as a compensatory mechanism. The problem is that excessive myocardial uptake of FA causes cardiac lipotoxicity and enhanced oxidative stress. The end result is diabetic cardiomyopathy, which is characterized by concentric left ventricular hypertrophy, abnormal diastolic function and reduced output. There is also an upregulation of peroxisome proliferator-activated receptor alpha (PPAR-α), which enhances FA oxidation [18]. Therefore, cardiac metabolism becomes highly altered. 

In addition, another proposed pathophysiological mechanism for myocardial dysfunction in diabetes leading to the development of diabetic cardiomyopathy is myocardial stiffness, resulting from cellular and extracellular matrix stiffness as well as cell-matrix interactions. Being the intrinsic cardiomyocyte stiffness its major contributor as a result of the impairment in its cytoskeleton. Several mechanisms are also involved in myocardial stiffness: inflammation, oxidative stress and SGLT-2 mediated effects, the reason why SGLT2i are emerging as a potential treatment option [19].

In summary, there seem to be two different metabolic disturbances that account for the failing heart: HF (which increased glucose metabolism and reduced FA utilization) and diabetic cardiomyopathy (with greater metabolic reliability on FA oxidation but lower glucose consumption). These two mechanisms suggest the existence of a very precise balance of different substrate consumption to ensure adequate cardiac function.

### 2.3. Myocardial Schemia: Metabolic Alterations

Following acute myocardial ischemia, a wound repair process of the left ventricle is triggered. This phenomenon, also termed “adverse remodeling”, involves changes such as myocyte death and hypertrophy, inflammatory response and connective tissue alterations. The extent of these changes is a major determinant of mortality and morbidity in patients with acute myocardial infarction [20,21]. In addition, reperfusion-induced molecular alterations may also contribute to the final extension of the cardiac injury [22,23].

Myocardial ischemia triggers a local and systemic inflammatory response in which both cytokines and cardiac-monomeric C-reactive protein seem to play a crucial role [24]. In addition, the activation of the innate inflammatory response via toll-like receptor 4 (TLR-4) may regulate this response [22]. Moreover, reperfusion-associated injury induces an upregulation of pro-fibrotic cytokine transforming growth factor beta (TGF-β) promoting fibrotic deposition, and some studies suggest that apoptosis is a reperfusion-triggered phenomenon and that is related to recruited macrophages [24]. The final result is a decrease in mitochondrial oxidative metabolism and ATP production [25]. Therefore, during myocardial ischemia, energy supply from oxidative metabolism (either from glucose or FA oxidation) cannot satisfy energy demands, and glycolysis becomes the only metabolic pathway available for energy production and cell survival. Nonetheless, this anaerobic pathway is less energy efficient and generates fewer ATP molecules [17] as mentioned previously, and also, it leads to the accumulation of deleterious products within cardiac cells (lactate and protons) [25]. In summary, the higher reliability on glycolysis during cardiac ischemia induces contractile reserve impairment, left ventricle hypertrophy and contractile dysfunction if it prolongs enough.

It is also important to highlight the difference between acute and chronic alterations after cardiac ischemia. Following acute ischemia, there is an upregulation of carbohydrates metabolism and glucose intake, which may be beneficial in early stages since glucose is a more oxygen efficient metabolite than FA [17]. However, a cardiac metabolism mostly dependable on glucose utilization may alter cardiac function in the long term, as observed in glycogen storage disease-related cardiomyopathy [26]. 

## 3. The Role of SGLT1 Receptor in Ischemic Cardiomyopathy

### 3.1. SGLT-1 Receptors Overview

Given its hydrophilic nature, glucose molecules are unable to pass the lipid bilayer of cardiac cells. Two families of transporters are involved in facilitate the transport of glucose from the bloodstream into the myocyte cytosol: 1) the glucose transporters 1 and 4 (GLUT1 and GLUT4), which transport exclusively glucose molecules via facilitated-diffusion and 2) the sodium-glucose cotransporter 1 (SGLT1), which transports various substrates into the cell (i.e., sugars, inositol and urea) against chemical gradient using the energy related in the simultaneous transport of sodium in favor of electrochemistry [25,27]. While GLUT1/4 has been widely investigated, little is yet known about SGLT1. Apart from the cardiomyocytes, SGLT1 receptors are also expressed in small intestine enterocytes and in renal proximal tubule S3 cells, wherein it also mediates glucose uptake [27]. 

The SLGT family is the subgroup solute carrier 5 (SLC5) of the solute carrier’s group. Among them, SGLT1 and SGLT2, encoded by the genes *SLC5A1* and *SLC5A2*, respectively, are of essential importance for glucose homeostasis [28]. Therefore, these receptors represent a potential target for treating diabetic patients and they have drawn tremendous attention over the last years. Interestingly and in contrast to SGLT2 receptor, that is mainly expressed in the kidneys, SGLT1 receptor is highly expressed in the human heart, with preferential location in the sarcolemma [11]. Its expression is also altered in diabetic and ischemic cardiomyopathy and it may be regulated by leptin [27]. While leptin stimulates the expression of cardiac SGLT1 mRNA, insulin seems to only stimulate its translocation to sarcolemma. Interestingly, at least in mice, cardiac SGLT1 expression has found to increase with age [27]. 

Functional SGLT1 is an oligomer, resulting from dimerization. At least 3 bands have been observed in human hearts: 2 bands of 70 and 140 kDa (that could reflect dimerization), and also an intermediate band, which its significance is unknown and could be related to post-translational modifications (i.e., phosphorylation) [27]. The expression of SGLT1 has been also found rat endothelial cells in skeletal muscle, brain and coronary arteries, where in is required for the action of insulin on glucose supply to myocytes [29]. However, this has not yet been confirmed in human endothelial cells [11]. Unlike GLUT1 and GLUT4, which expression is down-regulated in diabetic hearts, SGLT1 expression is increased in individuals with end-stage cardiomyopathy secondary to T2DM and in obese mice [27]. In contrast, SGLT1 expression decreases in a mice model with T1DM [27]. It has been postulated that increased SGLT1 expression may be related to chronic hyperinsulinaemia in T2DM and/or and adaptive response to reduced GLUT1 and GLUT4 expression [27]. Insulin activates protein kinase C and phosphorylation of SGLT1, which increases the recruitment of SGLT1 transporter to the plasma membrane and thus, glucose uptake. 

Intestinal SGLT1 is modulated by dietary carbohydrate consumption. Following a high-glucose diet in animal models, SGLT1 activity and expression increase. Glucose appears to be a local rather than a systemic modulator since oral but not intravenous glucose administration enhances its expression [30]. 

### 3.2. Impact of SGLT1 Inhibition on Ischemic Heart Disease

Cardiac ischemia results in 2- to 3-fold increased glucose uptake and utilization [31], that seems to be protective at least partially, during acute injury. There is an upregulation of expression and translocation of GLUT1 and GLUT4 via PI3-K dependent manner and also through adenosine mono-phosphate activated protein kinase (AMPK) [32]. The phosphorylation (activation) of Akt by phosphatidylinositol 3-kinase (PI3K) increases GLUT1/4 expression and may explain the cardioprotective effect of Akt on myocardial ischemia [23]. AMPK is the cell energy sensor that regulates cellular homeostasis during cardiac impairment. Hyperactivating mutation of AMKP in mice is associated with increased expression/activity of SGLT1 [11]. In addition, SGLT1 is also upregulated 2- to 3-fold in myocardial ischemia [27] and seems to be an adaptive response to injury give its association with the functional recovery in failing hearts after left ventricular assist device insertion [32]. This upregulation of SGLT1 receptors appears to be related to the activation of intracellular second messengers, ERK1/2 and mTOR [11]. 

Whether SGLT1 receptors exert protective or deleterious effects has not been yet determined. On one site, SGLT1 expression may be beneficial following acute myocardial ischemic injury since they facilitate glucose uptake, which is the only source of ATP through anaerobic glycolysis during ischemia. 

On the other side, chronic SGLT1 overexpression has been demonstrated to cause a phenotype similar to glycogen-storage cardiomyopathy [33,34]. Importantly a recent article has demonstrated that SGLT1-knockdown mice model was associated with reduced oxidative stress, myocardial necrosis and infarct size following ischemia-reperfusion (I/R) injury [35]. Specifically, during ischemia AMPK upregulates SGLT1 through ERK, and SGLT1 interacts with EGFR which in turn increases PKC and Nox2 activity and oxidative stress. Therefore, SLGLT1 may represent a novel therapeutic target for mitigating ischemia—reperfusion injury for patients with ischemic heart disease and further trial should be addressed [36]. Nevertheless, acute treatment with dual SGLT1/SGLT2 inhibitors immediately after I/R injury has also been associated with exacerbated cardiac dysfunction in rats [37]. Given the absence of SGLT2 receptors in cardiac cells, this effect may be linked to acute SGLT1 inhibition in the post-myocardial infarct setting. Moreover, I/R models treated with phlorizin, a selective SGLT1 inhibitor, also showed poor outcomes with increased infarct size and cardiac dysfunction [38]. 

In summary, the metabolic switch towards carbohydrate over FA consumption, following acute myocardial ischemia, seems to be essential to maintain adequate ATP supply to the cardiac contractile apparatus. SGLT1 receptors favor this glucose utilization, and SGLT1 inhibition may not be beneficial in the acute scenario. However, a long-term cardiac metabolism mainly based on glucose consumption has also been associated with cardiac impairment and thus, SGLT1 inhibitors may play a more decisive role in this setting. 

## 4. Inhibition of SGLT2 Receptors

### 4.1. SGLT2 Receptor Overview

Due to poor control of diabetes even with the high number of medications available, new therapeutic interventions are still being sought to try and control this fearsome epidemic. Type 2 sodium glucose co-transporter receptor inhibitors (SGLT2i) are a new class of drug with a promising future. 

This receptor plays a key role in the tubular reabsorption of the urinary glucose. Kidneys filter glucose molecules that are subsequently reabsorbed in the proximal tubules back to the bloodstream. When the amount of glucose in the renal tubules surpasses 160–180 mg/dL (i.e., renal threshold), the kidneys are unable to reabsorb such a high quantity and glucose is eliminated through urine (i.e., glycosuria) [39]. Approximately 90% of the glucose is transported through the membrane of the proximal tubule by the SGLT2 receptor. There is an overexpression of SGLT2 receptors in diabetic patients, which increases glucose reabsorption and thus, glycaemia [40]. Mutations in the gene that encodes this receptor, *SLC5A2*, have been associated with glycosuria suggesting that by blocking this cotransporter the reabsorption of glucose from the renal tubules may be impaired and therefore reducing hyperglycemia [39]. 

This observation offers the nature response and settles the basics for an alternative treatment of DM patients. In terms of side effects, the most frequent and relevant is mild-moderate GI infections, more commonly in women with prior history of infections and post – menopause that resolved with standard treatment. Given that its effect is not related to increased insulin release, hypoglycemia was not seen in patients with T2DM or nondiabetic, presumably due to an increase in endogenous glucose production in the liver. In addition, the is a mild diuretic effect that may increase the risk of orthostatic hypotension, postural dizziness and dehydration specially in older patients and with combination of loop diuretics [5] (Table 1 summarizes the properties of the SGLT2 inhibitors).

### 4.2. Impact of SGLT2 Inhibition on Heart Failure and Diabetic Cardiomyopathy

The rapidly growing interest in cardiac metabolism and substrate flexibility continues to increase due to these new emergent drugs, SGLT2i [41]. These agents have showed in three large randomized clinical trials [5,6,7], to reduce mortality and HF hospitalizations in T2DM patients [42]. By impressive reduction in HF events, these trials also provide strong evidence for primary prevention of new onset HF among T2DM that should be further evaluated [43]. We have specifically demonstrated that the SGLT2 inhibitor empagliflozin ameliorate adverse cardiac remodeling and enhance systolic function in HF animals even in non-diabetic model [44]; in fact our findings have been later corroborated by other groups [45,46], demonstrating a cardio-protective effect in non- DM models with cardiomyopathy [47].

We postulated that glycosuria induced by SGLT2 inhibition lowers portal insulin-to-glucagon ratio, which causes lipolysis and increased FFA delivery to the liver, thus resembling prolonged fasting and stimulating ketogenesis As myocardial ketone uptake is only dependent on plasma ketone concentrations and ketonemia is markedly increase during SGLT2 inhibition (both in diabetic and not diabetic patients) [48], we postulated [49] that empagliflozin would result in a metabolic switch in myocardial metabolism from glucose utilization (which is energy-inefficient) towards the consumption of ketone bodies and FA (which produce more energy compared with glucose). In fact, our results demonstrate reduced consumption of glucose but enhanced utilization of ketone bodies, FA and branched-chain aminoacids (BCAA) [49]. Furthermore, there was increased activity of all the enzymes responsible for FA/ketone/BCAA metabolism and reduced expression of enzymes implicated in glucose metabolism [49]. This metabolic switch has later been confirmed by other groups [45]. Moreover, the positive mechanistic role of ketone bodies has been further confirmed by us given that a continuous infusion of β-hydroxybutyrate improves cardiac function (both systolic and diastolic) in a non-diabetic animal model [50,51]. 

Additionally, SGLT2i inhibit the sodium-hydrogen exchanger (NHE) isoform 1, which is expressed in the myocardium and modulates cardiomyocyte pH. There is an upregulation of NHE1 activity in the failing heart, which leads to an increase in intracellular Ca^2+^ concentration, via promoting Na^+^ influx. As a consequence, calcineurin signaling is enhanced resulting in cell death and myocardial damage [17]. Long-term suppression of NHE1 in animals has been associated with reduced oxidative stress and thus, myocardial fibrosis and left ventricular adverse remodeling [52]. Moreover, NHE1 downregulation has been also related to preservations of insulin sensitivity in high-fat diet fed mice [52]. Importantly, SGLT2 inhibitors inhibit NHE [53,54] which may also explain the beneficial effects of these drugs on HF.

Cardiac fibrosis, an important final common pathway of HF, is also ameliorated by SGLT2 inhibition. On DM murine models after 8 weeks of empagliflozin myocardial structure and function improved showing a reduction on myocardial oxidative stress in addition of ameliorated fibrosis [55]. Dapagliflozin for instance, reduces collagen synthesis in rats by inhibiting myofibroblast differentiation following acute MI [56], and suppresses prolonged ventricular repolarization through increase mitochondrial function in insulin—resistant metabolic rats [57]. Empagliflozin also lowers pro-fibrotic markers via downregulation the cardiac fibroblast activation [58]. 

Another interesting postulated mechanism by which SGLT2i may exert their cardiac benefits is via AMPK activation. This protein-kinase promotes FA metabolism and oxidation and therefore, improving cardiac bioenergetics [59]. Moreover, SGLT2 inhibitors seem to polarize macrophages from a proinflammatory M1 phenotype towards an anti-inflammatory M2 phenotype [60], which would also cause less cardiac damage. 

A tight glycemic control takes years to display changes in CV outcomes and this is unlikely to be the reason behind the previously mentioned benefits. Interestingly, SGLT2 receptors are not expressed in the heart and that suggests an indirect effect on cardiac metabolism that remains still unknown [61]. Therefore, many research projects are now focusing on clarifying SGLT2i properties beyond DM, and more focalized around cardiac metabolism, cardiovascular effects (including arterial stiffness and vascular resistance) and a direct natriuretic impact with subsequent impact on blood pressure control [62,63].

SGLT2 inhibitors additionally affect intra cellular Ca^2+^ metabolism. Overexpression and Ca^2+^ dependent activation of Ca^2+^/calmodulin dependent kinase II CaMKII are hallmarks of HF. Empagliflozin reduces CaMKII activity and CaMKII dependent Ca^2+^ leak from the sarcoplasmic reticulum, which may contribute to the beneficial effect in HF [64]. SGLT2 inhibitors also affect intracellular concentration of Na^+^. Elevated cardiac cytoplasmic Na^+^ concentration is a driver of HF. By inhibiting NHE, SGLT2i directly lower cytoplasmic Na^+^ concentration in the cardiomyocyte [65,66]. 

### 4.3. SGLT2 Inhibitors and Clinical Outcomes 

A Significant number of effective drugs for long-term control of high-glucose levels in T2DM patients have demonstrated their effectiveness in reducing microvascular complications. Unfortunately, these drugs have not shown clear benefits in reducing CV adverse events, even few of them may be harmful when administered to patients with concomitant cardiac disease. The SGLT2i represent a new class of drug with a promising future. Recent large randomized controlled trials investigating safety outcomes with these agents have showed surprising cardiovascular benefits and even a significant mortality rate reduction. Furthermore, these drugs have also gained great attention due to their unclear mechanism of action that seems to be independently of their hypoglycemic effects, and whether is a drug-specific or generalizable to the drug class [65]. 

As a consequence of all the data reviewed in this article, SGLT2 inhibitors have evolved from antidiabetic treatments towards being studied also for HF, both in diabetic and in non-diabetic patients. The EMPEROR clinical trial is an event-driven trial that is studying this potential benefits. Building up on this hypothesis, we have initiated the EMPA-TROPISM (Are the “Cardiac Benefits” of Empagliflozin Independent of Its Hypoglycemic Activity?) (NCT03485222) clinical trial to investigate the effects of empagliflozin specifically in nondiabetic HF [12], while other event-driven large clinical trials such as EMPEROR-REDUCED (Empagliflozin outcome trial in Patients With chronic HF with Reduced Ejection Fraction) (NCT03057977) or EMPEROR-PRESERVED (Empagliflozin outcome trial in patients with chronic HF with preserved ejection fraction) (NCT03057951) also include HF patients independently of diabetic status. 

Additional clinical benefits have been observed in renal patients. Canagliflozin for instance, has demonstrated to mitigate kidney dysfunction and to attenuate eGFR decline and albuminuria, that suggests also a nephroprotective effect of this drug [66]. (Table 2 represents a comparison between SGLT1 and SGLT2 receptors).

## 5. Conclusions

The SGLT receptors have drawn a major interest following the surprising results of the EMPA-REG OUTCOME trial [5]. Its role beyond only treating patient with DM has been undoubtedly established. Unlike SGLT2, SGLT1 receptors are expressed in human hearts and they seem to play a major role via favoring carbohydrate metabolism. This phenomenon is certainly important in the acute phase of I/R injury but appears to be detrimental if it prolongs, such as in patients with chronic HF. 

In addition, SLGT1 but not SGLT2 have demonstrated to exert pro-inflammatory properties that may intensify myocardial infarct size and contractile dysfunction. Whether SGLT1 inhibition contributes to reduce this phenomenon and provide better outcomes after I/R injury remains still unclear.

Interestingly, SGLT2 inhibitors but not SGLT1 have demonstrated to promote KB metabolism and therefore, to enhance cardiac bioenergetics. This circumstance is of particular interest in patients with chronic damaged hearts. Whether this effect could be strengthened by a dual SGTL1 and SGLT2 inhibition should also be further investigated.

## Figures and Tables

**Table 1:** Pharmacological properties of most common used SGLT inhibitors and clinical outcomes.

SGLT2 Inhibitor	Bioavailability	Time-to Peak/Half-Life	Excretion	Initial (Max) Dose	Clinical Trials	CV Outcomes	Renal Outcome	Effects	Main Adverse Effects
Empagliflozin [5]	~75%	1.5 h/13 h	55% Renal 40% Fecal	10 mg (25 mg)	EMPA-REG OUTCOME 10 or 25 mg for 3.1 years	Reduced CV death (RRR of 38%) Reduced hospitalization for HF (RRR 35%) Reduced death from any cause (RRR 32%)	Reduced progression of kidney disease (RRR 44% of doubled creatinine)	Pancreatic β-cell function improvement Weight loss Natriuretic effect Decreased SBP Decreased Acid uric Small increase in HDL-c and LDL-c	GTI Hypoglycemia Infrequent
Canagliflozin [6]	~65%	1–2 h/13 h	41.5% Fecal 33% Renal	100 mg (300 mg)	CANVAS 100 or 300 mg for 3.6 years	Reduced hospitalization for HF (RRR 33%) Reduced death from any cause (RRR 13%)	Reduced progression of kidney disease (RRR 27%, albuminuria progression)	Weight loss Natriuretic effect Decreased SBP Decreased Acid uric Small increase in HDL-c and LDL-c	GTI Hypoglycemia (more common than dapagliflozin/ empagliflozin) May ↑ lower extremities amputations
Dapagliflozin [7]	~78%	1–1.5 h/13 h	75% Renal 21% Fecal	5 mg (10 mg)	DECLARE-TIMI38 10 mg for 4.2 years DAPA-HF 5 or 10 mg for 3 years	No yet available	May reduce progression of kidney disease (scarce data)	Weight loss Natriuretic effect Decreased SBP Decreased Acid uric Small increase in HDL-c and LDL-c	GTI Hypoglycemia Headache Diarrhea May increase breast and bladder cancer rates
Ertugliflozin [37]	~70–90%	0.5–1.5 h/11–17 h	50% Renal 41% Fecal	5 mg (15 mg)	VERTIS-CV 5 or 15 mg for 6.1 years	No yet available	Not yet available	Weight loss Natriuretic effect Decreased SBP Decreased Acid uric Small increase in HDL-c and LDL-c	GTI Hypoglycemia
Sotagliflozin (dual SGLT 1 and 2 inhibitor) [41]	-	3 h/13.5–20.7 h	Mostly Renal	200 mg (400 mg)	inTANDEM 400 mg for 24 weeks	Not yet available	Not yet available	Under investigation	GTI Nausea/Diarrhea

CV: cardiovascular; GTI: Genitourinary tract infections; HDL-c: high-density lipoprotein cholesterol; HF: heart failure; LDL-c: low-density lipoprotein cholesterol; RRR: relative risk reduction; SBP: systolic blood pressure; SGLT: sodium-glucose cotransporter.

**Table 2:** Comparison of SGLT1 versus SGLT2 inhibitors.

Characteristic	SGLT1	SGLT2
Capacity	Low	High
Affinity	High	Low
Function	Dietary absorption glucose and galactose (GIT) Renal reabsorption glucose	Renal reabsorption glucose
Renal location	S3 of PCT	S1 and S2 of PCT
Renal glucose reabsorption	10%	90%
Ratio Na-Glucose cotransport	2:1	1:1
Gene encoding	*SCL5A1*	*SLC5A2*

GIT: gastrointestinal tract, PCT: proximal convoluted tubule; SGLT: sodium-glucose cotransporter.

## References

[1] Beer M., Seyfarth T., Sandstede J., Landschütz W., Lipke C., Köstler H., Von Kienlin M., Harre K., Hahn D., Neubauer S. (2002). Absolute concentrations of high-energy phosphate metabolites in normal, hypertrophied, and failing human myocardium measured noninvasively with31P-SLOOP magnetic resonance spectroscopy. J. Am. Coll. Cardiol..

[2] Kolwicz S.J., Olson D., Marney L., Garcia-Menendez L., Synovec R., Tian R. (2012). Cardiac-specific deletion of acetyl CoA carboxylase 2 prevents metabolic remodeling during pressure-overload hypertrophy. Circ. Res..

[3] Zhou Y.-T., Grayburn P., Karim A., Shimabukuro M., Higa M., Baetens D., Orci L., Unger R.H. (2000). Lipotoxic heart disease in obese rats: Implications for human obesity. Proc. Natl. Acad. Sci. USA.

[4] Abubakar I.I., Tillmann T., Banerjee A. (2015). Global, regional, and national age-sex specific all-cause and cause-specific mortality for 240 causes of death, 1990–2013: A systematic analysis for the Global Burden of Disease Study 2013. Lancet.

[5] Zinman B., Wanner C., Lachin J.M., Fitchett D., Bluhmki E., Hantel S., Mattheus M., Devins T., Johansen O.E., Woerle H.J. (2015). Empagliflozin, Cardiovascular Outcomes, and Mortality in Type 2 Diabetes. N. Engl. J. Med..

[6] Neal B., Perkovic V., Mahaffey K.W., de Zeeuw D., Fulcher G., Erondu N., Shaw W., Law G., Desai M., Matthews D.R. (2017). Canvas. N. Engl. J. Med..

[7] Wiviott S.D., Raz I., Bonaca M.P., Mosenzon O., Kato E.T., Cahn A., Silverman M.G., Zelniker T.A., Kuder J.F., Murphy S.A. (2018). Dapagliflozin and Cardiovascular Outcomes in Type 2 Diabetes. N. Engl. J. Med..

[8] Flores E., Santos-Gallego C.G., Diaz-Mejía N., Badimon J.J. (2018). Do the SGLT-2 Inhibitors Offer More than Hypoglycemic Activity?. Cardiovasc. Drugs Ther..

[9] Wanner C., Inzucchi S.E., Lachin J.M., Fitchett D., von Eynatten M., Mattheus M., Johansen O.E., Woerle H.J., Broedl U.C., Zinman B. (2016). Empagliflozin and Progression of Kidney Disease in Type 2 Diabetes. N. Engl. J. Med..

[10] Perkovic V., Jardine M.J., Neal B., Bompoint S., Heerspink H.J., Charytan D.M., Edwards R., Agarwal R., Bakris G., Bull S. (2019). Canagliflozin and Renal Outcomes in Type 2 Diabetes and Nephropathy. N. Engl. J. Med..

[11] Di Franco A., Cantini G., Tani A., Coppini R., Zecchi-Orlandini S., Raimondi L., Luconi M., Mannucci E. (2017). Sodium-dependent glucose transporters (SGLT) in human ischemic heart: A new potential pharmacological target. Int. J. Cardiol..

[12] Santos-Gallego C.G., Garcia-Ropero A., Mancini D., Pinney S.P., Contreras J.P., Fergus I., Abascal V., Moreno P., Atallah-Lajam F., Tamler R. (2019). Rationale and Design of the EMPA-TROPISM Trial (ATRU-4): Are the “Cardiac Benefits” of Empagliflozin Independent of its Hypoglycemic Activity?. Cardiovasc. Drugs Ther..

[13] Palomer X., Salvadó L., Barroso E., Vázquez-Carrera M. (2013). An overview of the crosstalk between inflammatory processes and metabolic dysregulation during diabetic cardiomyopathy. Int. J. Cardiol..

[14] Wende A.R., Brahma M.K., McGinnis G.R., Young M.E. (2017). Metabolic Origins of Heart Failure. JACC Basic Transl. Sci..

[15] Doenst T., Nguyen T.D., Abel E.D. (2013). Cardiac Metabolism in Heart Failure. Circ. Res..

[16] Noordali H., Loudon B.L., Frenneaux M.P., Madhani M. (2018). Cardiac metabolism—A promising therapeutic target for heart failure. Pharmacol. Ther..

[17] Garcia-Ropero A., Santos-Gallego C.G., Zafar M.U., Badimon J.J. (2019). Metabolism of the failing heart and the impact of SGLT2 inhibitors. Expert Opin. Drug Metab. Toxicol..

[18] Maack C., Lehrke M., Backs J., Heinzel F.R., Hulot J.-S., Marx N., Paulus W.J., Rossignol P., Taegtmeyer H., Bauersachs J. (2018). Heart failure and diabetes: Metabolic alterations and therapeutic interventions: A state-of-the-art review from the Translational Research Committee of the Heart Failure Association–European Society of Cardiology. Eur. Heart J..

[19] Nikolajević Starčević J., Janić M., Šabovič M. (2019). Molecular Mechanisms Responsible for Diastolic Dysfunction in Diabetes Mellitus Patients. Int. J. Mol. Sci..

[20] Pfeffer M., Braunwald E. (1990). Ventricular Remodeling After Myocardial Infarction. Circulation.

[21] Gajarsa J.J., Kloner R.A. (2011). Left ventricular remodeling in the post-infarction heart: A review of cellular, molecular mechanisms, and therapeutic modalities. Heart Fail. Rev..

[22] Sun Y. (2009). Myocardial repair/remodelling following infarction: Roles of local factors. Cardiovasc. Res..

[23] Santos-Gallego C.G., Vahl T.P., Goliasch G., Picatoste B., Arias T., Ishikawa K., Njerve I.U., Sanz J., Narula J., Sengupta P.P. (2016). Sphingosine-1-Phosphate Receptor Agonist Fingolimod Increases Myocardial Salvage and Decreases Adverse Postinfarction Left Ventricular Remodeling in a Porcine Model of Ischemia/Reperfusion. Circulation.

[24] Vilahur G., Juan-Babot O., Peña E., Oñate B., Casaní L., Badimon L. (2011). Molecular and cellular mechanisms involved in cardiac remodeling after acute myocardial infarction. J. Mol. Cell. Cardiol..

[25] Dyck J.R.B., Lopaschuk G.D. (2006). AMPK alterations in cardiac physiology and pathology: Enemy or ally?. J. Physiol..

[26] Goodyear L.J., Xing Y., Wolf C., Bali D., Perez-Atayde A.R., He H., Berul C.I., Tian R., Stapleton D., Ahmad F. (2005). Increased α2 Subunit–Associated AMPK Activity and PRKAG2 Cardiomyopathy. Circulation.

[27] Banerjee S.K., McGaffin K.R., Pastor-Soler N.M., Ahmad F. (2009). SGLT1 is a novel cardiac glucose transporter that is perturbed in disease states. Cardiovasc. Res..

[28] Song P., Onishi A., Koepsell H., Vallon V. (2016). Sodium glucose cotransporter SGLT1 as a therapeutic target in diabetes mellitus. Expert Opin. Ther. Targets.

[29] Elfeber K., Stümpel F., Gorboulev V., Mattig S., Deussen A., Kaissling B., Koepsell H. (2004). Na^+^-D-glucose cotransporter in muscle capillaries increases glucose permeability. Biochem. Biophys. Res. Commun..

[30] Poulsen S., Fenton R., Rieg T. (2015). Sodium-glucose cotransport. Curr. Opin. Nephrol. Hypertens..

[31] Young L., Coven D., Russell R. (2000). Cellular and molecular regulation of cardiac glucose transport. J. Nucl. Cardiol..

[32] Szablewski L. (2017). Glucose transporters in healthy heart and in cardiac disease. Int. J. Cardiol..

[33] Banerjee S.K., Wang D.W., Alzamora R., Huang X.N., Pastor-Soler N.M., Hallows K.R., McGaffin K.R., Ahmad F. (2010). SGLT1, a novel cardiac glucose transporter, mediates increased glucose uptake in PRKAG2 cardiomyopathy. J. Mol. Cell. Cardiol..

[34] Ramratnam M., Sharma R.K., D’Auria S., Lee S.J., Wang D., Huang X.Y.N., Ahmad F. (2014). Transgenic Knockdown of Cardiac Sodium/Glucose Cotransporter 1 (SGLT1) Attenuates PRKAG2 Cardiomyopathy, Whereas Transgenic Overexpression of Cardiac SGLT1 Causes Pathologic Hypertrophy and Dysfunction in Mice. J. Am. Heart Assoc..

[35] Jakubiak M., Agrawal V., D’Auria S., Li Z., Ramratnam M., Sincoular A., Sharma R.K., Music M.L., Gifford L., Huang X.N. (2019). Cardiac Sodium-Glucose Co-Transporter 1 (SGLT1) is a Novel Mediator of Ischemia/Reperfusion Injury. Cardiovasc. Res..

[36] Garcia-Ropero A., Santos-Gallego C.G., Badimon J.J. (2019). SGLT receptors and myocardial ischaemia-reperfusion injury: Inhibition of SGLT-1, SGLT-2, or both?. Cardiovasc. Res..

[37] Connelly K.A., Zhang Y., Desjardins J.-F., Thai K., Gilbert R.E. (2018). Dual inhibition of sodium–glucose linked cotransporters 1 and 2 exacerbates cardiac dysfunction following experimental myocardial infarction. Cardiovasc. Diabetol..

[38] Kashiwagi Y., Nagoshi T., Yoshino T., Tanaka T.D., Ito K., Harada T., Takahashi H., Ikegami M., Anzawa R., Yoshimura M. (2015). Expression of SGLT1 in Human Hearts and Impairment of Cardiac Glucose Uptake by Phlorizin during Ischemia-Reperfusion Injury in Mice. PLoS ONE.

[39] Clar C., Gill J.A., Court R., Waugh N. (2012). Systematic review of SGLT2 receptor inhibitors in dual or triple therapy in type 2 diabetes. BMJ Open.

[40] Garcia-Ropero A., Badimon J.J., Santos-Gallego C.G. (2018). The pharmacokinetics and pharmacodynamics of SGLT2 inhibitors for type 2 diabetes mellitus: The latest developments. Expert Opin. Drug Metab. Toxicol..

[41] Xu L., Nagata N., Nagashimada M., Zhuge F., Ni Y., Chen G., Mayoux E., Kaneko S., Ota T. (2017). SGLT2 Inhibition by Empagliflozin Promotes Fat Utilization and Browning and Attenuates Inflammation and Insulin Resistance by Polarizing M2 Macrophages in Diet-induced Obese Mice. EBioMedicine.

[42] Santulli G. (2019). Editorial: Cardiovascular Disease and Diabetes. Front. Endocrinol..

[43] Tanaka A., Node K. (2018). Exploration of the clinical benefits of sodium glucose co-transporter 2 inhibitors in diabetic patients with concomitant heart failure. Cardiovasc. Diabetol..

[44] Greene S.J., Butler J. (2019). Primary Prevention of Heart Failure in Patients with Type 2 Diabetes Mellitus. Circulation.

[45] Santos-Gallego C., Requen Ibañez J., San Antonio R., Picatoste B., Watanabe S., Ishikawa K., Flores E., Garcia-Ropero A., Sanz J., Hajjar R. (2019). Empagliflozin Ameliorates Adverse LV Remodeling in a Non-Diabetic Model of Heart Failure Mediated via a Switch in Myocardial Metabolism That Enhances Energetics. J. Am. Coll. Cardiol..

[46] Yurista S.R., Silljé H.H., Oberdorf-Maass S.U., Schouten E., Pavez Giani M.G., Hillebrands J., van Goor H., van Veldhuisen D.J., de Boer R.A., Westenbrink B.D. (2019). Sodium–glucose co-transporter 2 inhibition with empagliflozin improves cardiac function in non-diabetic rats with left ventricular dysfunction after myocardial infarction. Eur. J. Heart Fail..

[47] Baker H.E., Kiel A.M., Luebbe S.T., Simon B.R., Earl C.C., Regmi A., Roell W.C., Mather K.J., Tune J.D., Goodwill A.G. (2019). Inhibition of sodium–glucose cotransporter-2 preserves cardiac function during regional myocardial ischemia independent of alterations in myocardial substrate utilization. Basic Res. Cardiol..

[48] Takasu T., Takakura S. (2019). Effect of ipragliflozin, an SGLT2 inhibitor, on cardiac histopathological changes in a non-diabetic rat model of cardiomyopathy. Life Sci..

[49] Ferrannini E., Mark M., Mayoux E. (2016). CV Protection in the EMPA-REG OUTCOME Trial: A “Thrifty Substrate” Hypothesis. Diabetes Care.

[50] Santos-Gallego C.G., Ibanez J.A.R., Antonio R.S., Ishikawa K., Watanabe S., Picatoste Botija M.B., Salvo A.J.S., Hajjar R., Fuster V., Badimon J. (2018). Empagliflozin Induces a Myocardial Metabolic Shift from Glucose Consumption to Ketone Metabolism That Mitigates Adverse Cardiac Remodeling and Improves Myocardial Contractility. J. Am. Coll. Cardiol..

[51] SantosGallego C.G., Requena-Ibanez J.A., San Antonio R., Ishikawa K., Picatoste B., Garcia-Ropero A., Sanz J., Hajjar R., Fuster V., Badimon J.J. (2018). Abstract 17367: Infusion of the Ketone Body β-Hydroxybutyrate Improves Left Ventricular Systolic Function in an Animal Model of Heart Failure with Reduced Ejection Fraction. Circulation.

[52] Prasad V., Lorenz J., Miller M., Vairamani K., Nieman M., Wang Y., Shull G. (2013). Loss of NHE1 activity leads to reduced oxidative stress in heart and mitigates high-fat diet-induced myocardial stress. J. Mol. Cell. Cardiol..

[53] Baartscheer A., Schumacher C.A., Wüst R.C., Fiolet J.W., Stienen G.J., Coronel R., Zuurbier C.J. (2017). Empagliflozin decreases myocardial cytoplasmic Na^+^ through inhibition of the cardiac Na^+^/H^+^ exchanger in rats and rabbits. Diabetologia.

[54] Uthman L., Baartscheer A., Bleijlevens B., Schumacher C.A., Fiolet J.W., Koeman A., Jancev M., Hollmann M.W., Weber N.C., Coronel R. (2018). Class effects of SGLT2 inhibitors in mouse cardiomyocytes and hearts: Inhibition of Na^+^/H^+^ exchanger, lowering of cytosolic Na+ and vasodilation. Diabetologia.

[55] Li C., Zhang J., Xue M., Li X., Han F., Liu X., Xu L., Lu Y., Cheng Y., Li T. (2019). SGLT2 inhibition with empagliflozin attenuates myocardial oxidative stress and fibrosis in diabetic mice heart. Cardiovasc. Diabetol..

[56] Lee T.M., Chang N.C., Lin S.Z. (2017). Dapagliflozin, a selective SGLT2 Inhibitor, attenuated cardiac fibrosis by regulating the macrophage polarization via STAT3 signaling in infarcted rat hearts. Free Radic. Biol. Med..

[57] Durak A., Olgar Y., Degirmenci S., Akkus E., Tuncay E., Turan B. (2018). A SGLT2 inhibitor dapagliflozin suppresses prolonged ventricular-repolarization through augmentation of mitochondrial function in insulin-resistant metabolic syndrome rats. Cardiovasc. Diabetol..

[58] Kang S., Verma S., Teng G., Belke D., Svystonyuk D., Guzzardi D., Park D., Turnbull J., Malik G., Fedak P. (2017). Direct effect of empagliflozin on extracellular matrix remodeling in human cardiac fibroblasts: Novel translational clues to EMPA-REG OUTCOME. Can. J. Cardiol..

[59] Danne T., Biester T., Kordonouri O. (2018). Combined SGLT1 and SGLT2 Inhibitors and Their Role in Diabetes Care. Diabetes Technol. Ther..

[60] Miller M., Genuth S., Ismail-Beigi F., Buse J., Goff J., Probstfield J., Cushman W., Ginsberg H., Bigger J., ACCORD Study Group (2011). Long-term effects of intensive glucose lowering on cardiovascular outcomes. N. Engl. J. Med..

[61] Cherney D.Z.I., Perkins B.A., Soleymanlou N., Har R., Fagan N., Johansen O.E., Woerle H.J., von Eynatten M., Broedl U.C. (2014). The effect of empagliflozin on arterial stiffness and heart rate variability in subjects with uncomplicated type 1 diabetes mellitus. Cardiovasc. Diabetol..

[62] Anker S.D., Butler J. (2018). Empagliflozin, calcium, and SGLT1/2 receptor affinity: Another piece of the puzzle. ESC Heart Fail..

[63] Mustroph J., Wagemann O., Lücht C.M., Trum M., Hammer K.P., Sag C.M., Lebek S., Tarnowski D., Reinders J., Perbellini F. (2018). Empagliflozin reduces Ca/calmodulin-dependent kinase II activity in isolated ventricular cardiomyocytes. ESC Heart Fail..

[64] Lee T.-I., Chen Y.-C., Lin Y.-K., Chung C.-C., Lu Y.-Y., Kao Y.-H., Chen Y.-J. (2019). Empagliflozin Attenuates Myocardial Sodium and Calcium Dysregulation and Reverses Cardiac Remodeling in Streptozotocin-Induced Diabetic Rats. Int. J. Mol. Sci..

[65] Bethel M.A., McMurray J.V. (2018). Class Effect for Sodium Glucose-Cotransporter-2 Inhibitors in Cardiovascular Outcomes. Circulation.

[66] Perkovic V., de Zeeuw D., Mahaffey K.W., Fulcher G., Erondu N., Shaw W., Barrett T.D., Weidner-Wells M., Deng H., Matthews D.R. (2018). Canagliflozin and renal outcomes in type 2 diabetes: Results from the CANVAS Program randomised clinical trials. Lancet Diabetes Endocrinol..

